# Effects of *Toxoplasma gondii* infection on cognition, symptoms, and response to digital cognitive training in schizophrenia

**DOI:** 10.1038/s41537-022-00292-2

**Published:** 2022-11-25

**Authors:** Anna Luiza Guimarães, David Richer Araujo Coelho, Linda Scoriels, Juliana Mambrini, Lis Ribeiro do Valle Antonelli, Priscilla Henriques, Andréa Teixeira-Carvalho, Olindo Assis Martins Filho, José Mineo, Lilian Bahia-Oliveira, Rogério Panizzutti

**Affiliations:** 1 Instituto de Psiquiatria, Universidade Federal do Rio de Janeiro, Rio de Janeiro, Brazil; 2 Instituto de Ciências Biomédicas, Universidade Federal do Rio de Janeiro, Rio de Janeiro, Brazil; 3 Departamento de Imunoparasitologia, Universidade Federal do Rio de Janeiro, Rio de Janeiro, Brazil; 4 Instituto René Rachou, Fundação Oswaldo Cruz, Rio de Janeiro, Brazil; 5 Universidade Federal de Uberlândia, Uberlândia, Brazil

**Keywords:** Schizophrenia, Learning and memory

## Abstract

Studies indicate that neuroscience-informed digital cognitive training can remediate cognitive impairments in schizophrenia, but the factors contributing to these deficits and response to treatment remain unclear. *Toxoplasma gondii* is a neuroinvasive parasite linked to cognitive decline that also presents a higher prevalence in schizophrenia. Here, we compared the cognition and symptom severity of IgG seropositive (TOXO+; *n* = 25) and seronegative (TOXO−; *n* = 35) patients who participated in a randomized controlled trial of digital cognitive training. At baseline, TOXO+ subjects presented lower global cognition than TOXO− (*F* = 3.78, *p* = 0.05). Specifically, TOXO+ subjects showed worse verbal memory and learning (*F* = 4.48, *p* = 0.03), social cognition (*F* = 5.71, *p* = 0.02), and higher antibody concentrations were associated with increased negative (*r* = 0.42, *p* = 0.04) and total (*r* = 0.40, *p* = 0.04) schizophrenia symptoms. After training, the TOXO+ group showed higher adherence to the intervention (*X*^2^ = 9.31, *p* = 0.03), but there were no differences in changes in cognition and symptoms between groups. These findings highlight the association between seropositivity to *T. gondii* and deteriorated cognition and symptoms in schizophrenia. Further research is needed to assess the specific efficacy of digital cognitive training on this population.

## Introduction

Schizophrenia is a complex debilitating neuropsychiatric disorder and its prevalence is estimated at approximately 1% of the world population^1,2^. Subjects with schizophrenia present a large range of symptoms, which significantly impact their quality of life, and about 85% of them have some degree of cognitive impairment^3^. The domains commonly affected are speed of processing, attention, working memory, verbal learning and memory, visual learning and memory, reasoning and problem solving, and social cognition^4,5^. The antipsychotic medication improves the positive symptoms of the disorder, but they lack efficiency for the cognitive impairments^6^.

Neuroscience-informed digital cognitive training has shown effectiveness in remediating cognitive deficits in schizophrenia^7–9^. However, response to this type of intervention has large variability, with effect sizes ranging from small to medium size^10^. More recent reviews suggest that future trials keep exploring moderators of response to cognitive interventions in schizophrenia^11,12^, such as biological mechanisms associated with the disorder.

The etiology of schizophrenia remains relatively unknown, although several risk factors have been identified. For instance, infectious agents, such as *Toxoplasma gondii (T. gondii)*, are environmental factors that significantly increase the risk of schizophrenia^13^. *T. gondii* is a neurotropic protozoan parasite, which infects approximately one-third of the global human population^14^, and may affect neural processing by forming cysts in the brain^15^. Meta-analyses have shown that subjects with schizophrenia are more likely to be seropositive for toxoplasmosis when compared with the general population (O.R. 2.73) (95% CI 2.10–3.60)^16^ and (OR 2.71) (95% CI 1.93–3.80)^17^. Seropositive subjects also show higher chances of dying of natural causes (O.R. 4.70) (95% CI 1.27–17.31)^18^. Additionally, studies have identified that women infected by *T. gondii* in their prenatal period present an increased risk of having offspring that will develop cognitive impairment and schizophrenia^19^. Moreover, children infected congenitally with *T. gondii* present lower IQ scores (93.2) compared with non-infected children of the same age (109.8)^20^. The parasite has also been associated with psychomotor impairments in subjects without psychiatric disorders^21^ and learning and memory deficits in mice^22^.

It is not clear how *T. gondii* infection affects individuals with schizophrenia, especially in terms of cognitive impairment and specific clinical symptoms associated with the disorder. Studies indicate a relationship between startle latency response and IgG titers in schizophrenia subjects and healthy controls^23^, and that toxoplasmosis in men with schizophrenia may lead to more severe negative and cognitive symptoms and a less favorable course of the disorder compared to non-infected male subjects^24^. But many studies did not find a significant relationship between seropositivity and cognitive impairment in schizophrenia^25–27^. Additionally, a birth cohort found a positive association between *T. gondii* infection and the prevalence of mental disorders, but not specifically schizophrenia^28^.

Considering the uncertain impact *T. gondii* infection may have on schizophrenia, we posed two questions: 1) Is *T. gondii* seropositivity associated with poorer cognitive performance and increased symptoms at baseline? 2) Could it result in different responses to neuroscience-informed digital cognitive training? To answer these questions, we measured antibody titers for *T. gondii* from individuals with schizophrenia who participated in a randomized, double-blind clinical trial on 40 h of neuroscience-informed cognitive training^7^.

## Results

### Baseline comparisons between TOXO+ and TOXO− schizophrenia subjects

Participants’ baseline characteristics are presented in Table 1. Concerning the serological profile, 58.3% of participants were IgG- (TOXO− group, *n* = 35), 41.7% were IgG + (TOXO+ group, *n* = 25), and all participants were IgM-, confirming no current infection. The mean age of participants in the TOXO+ group was significantly higher than the mean age in the TOXO− group (*X*^2^ = 4.09, *p* = 0.04), and we also detected a trend towards significance for more years of education (*t* = 1.70, *p* = 0.09) and higher Intelligence Quotient (*t* = 1.85, *p* = 0.07) in the TOXO+ group when compared to the TOXO−. Given the association between the Socioeconomic Status (SES) and both exposure to *T. gondii*^29^ and cognitive performance^30^, we measured this potentially confounding variable using the Social Development Index. We found no significant difference in the Social Development Index between TOXO+ and TOXO− groups (*X*^2^ = 1.72, *p* = 0.18).

**Table 1:** Participants’ baseline characteristics.

	All subjects (*n* = 60)	TOXO− (*n* = 35)	TOXO+ (*n* = 25)	Statistics	
	Median (SD or range)			Uncorrected t or X^2^ (*p*)^a^	Age-corrected β or F (*p*)^b^
Age (years)	42 (18–60)	36 (18–55)	45 (21–60)	**4.09 (0.04)**	
Female/male	18/44	10/27	8/17	0.17 (0.67)	
Education (years)	12 (4–19)	12 (7–19)	12 (4–16)	1.70 (0.09)	
IQ	100 (13)	106 (13)	95 (13)	*1.85 (0.07)*	
Years of Illness	14.5 (1–41)	13 (1–37)	18 (4–41)	−1.34 (0.19)	
CPZ equivalent (mg)	332 (406)	332 (350)	319 (488)	0.01 (0.91)	
Social Development Index	0.63 (0.10)	0.67 (0.10)	0.60 (0.10)	1.72 (0.18)	
Baseline cognition (z-scores)					
Speed of processing	−0.69 (2.28)	−0.37 (2.20)	−1.30 (2.37)	1.42 (0.23)	−0.19 (0.80)
Attention	−0.91 (1.41)	−0.76 (1.17)	−1.27 (1.56)	**2.41 (0.01)**	−0.55 (0.32)
Working memory	−0.34 (1.14)	−0.27 (1.25)	−0.54 (0.87)	1.63 (0.10)	1.91 (0.17)
Verbal memory and learning	−0.24 (1.15)	−0,13 (1.19)	−0.33 (0.97)	*3.26 (0.07)*	**4.48 (0.03)**
Visual memory and learning	−1.45 (1.49)	−1,13 (1.35)	−1.87 (1.64)	1.22 (0.22)	0.17 (0.67)
Reasoning and problem solving	0.15 (0.69)	0,15 (0.61)	0.11 (0.80)	0.03 (0.85)	−0.00 (0.99)
Social cognition	−0.28 (0.72)	0,05 (0.74)	−0.63 (0.59)	**2.52 (0.01)**	**5.71 (0.02)**
Global cognition	−0.70 (0.83)	−0.34 (0.78)	−1.00 (0.82)	**2.59 (0.01)**	**3.78 (0.05)**
Baseline clinical measures (range)					
HAM-D (0–54)	5 (5)	5 (5)	5 (6)	0.07 (0.78)	0.00 (1.00)
HAM-A (0–56)	4 (6)	5 (6)	4 (6)	0.13 (0.71)	1.00 (0.57)
PANSS Positive score (7–49)	13 (5)	13 (5)	13 (5)	0.00 (0.60)	0.19 (0.66)
PANSS Negative score (7–49)	15 (6)	16 (6)	15 (5)	0.98 (0.65)	0.32 (0.57)
PANSS General score (16–112)	28 (7)	29 (6)	28 (7)	0.02 (0.49)	0.38 (0.53)
PANSS Total Score (30–210)	57 (14)	57 (15)	56 (13)	0.82 (0.49)	0.45 (0.50)
Serological profile					
*T T. gondii* IgM titers^**c**^		6.05 (2.45–65.7)	8.57 (2.66–64.6)		
*T. gondii* IgG titers^**d**^		5.66 (0.71–19.2)	89.60 (22.2–99.6)		

^**a**^In the first column we compared TOXO+ and TOXO− groups using either *t*-test (two-tailed) for normal distributed variables (Years of illness, IQ and Education, Attention, working memory, spatial memory, social, and global cognition, PANSS Negative and Total) or Kruskal-Wallis equality-of-populations rank test for non-normal distributions (Age, CPZ, Social Development Index, Speed of processing, verbal memory, reasoning and problem solving, HAM-A, HAM-D, PANSS Positive and General).

^**b**^In the second column, General linear models were used to control for age within variables that respected homoscedasticity (Verbal Memory, Working Memory, Spatial Memory, Social Cognition, Global Cognition and all PANSS scores), and Quantile Regression was used to non-parametric models (Attention, Speed of Processing, Problem Solving, HAM-D and HAM-A).

^**c**^Positive reference > 80.

^**d**^Positive reference > 20.

*IQ* Intelligence quotient, *CPZ* Chlorpromazine, *HAM-D* Hamilton Depression Rating Scale, *HAM-A* Hamilton Anxiety Rating Scale, *PANSS* Positive and Negative Syndrome Scale.

Bold values show *p* values < 0.05.

Italic numbers trend towards significance.

#### Seropositivity to *T. gondii* and cognitive performance in schizophrenia

**Fig. 1:**
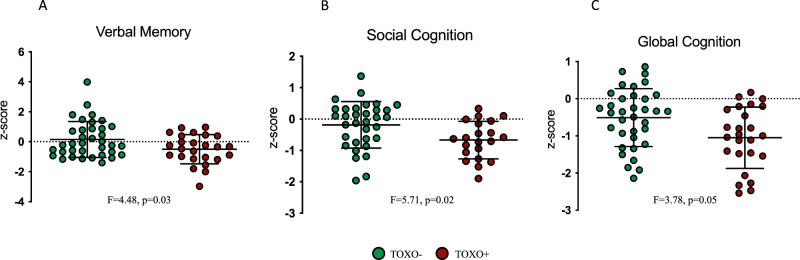
Differences in cognition between TOXO+ and TOXO− groups. The TOXO+ group had lower mean Z-scores in Verbal Memory (**A**), Social Cognition (**B**) and Global Cognition (**C**) when compared to the TOXO− group. Inserts show F and *p*-values of the general linear models using age as a covariate.

Baseline comparisons showed that TOXO+ subjects had significantly lower z-scores in attention, social and global cognition, and a trend towards significance for verbal memory and learning when compared to TOXO− subjects. After running a model including age as a covariate, differences between groups remained significant to social and global cognition, and the trend observed in the first analysis of verbal memory showed a significant p-value on the covariate model (Fig. 1). Nevertheless, after including age as a covariate the differences in attention were no longer significant. No significant group differences were found in Speed of Processing, Working Memory, Spatial Memory or Reasoning and Problem Solving (Table 1).

We next asked whether the IgG titers would be associated with cognitive performance in the TOXO+ group. We only found a trend towards significance for social cognition (*r* = −0.39, *p* = 0.07) (Supplementary Table 1).

#### Seropositivity to *T. gondii* and schizophrenia symptoms

**Fig. 2:**
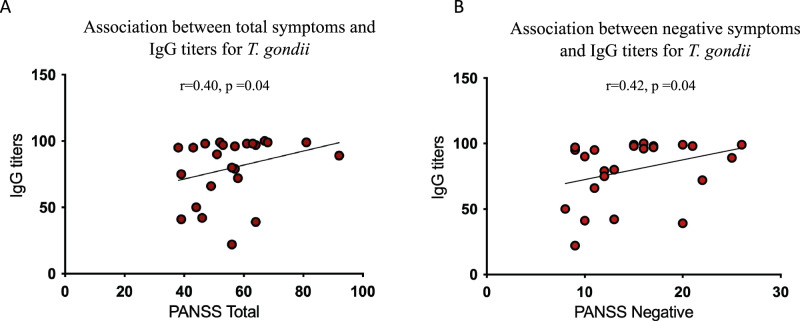
Spearman correlations between IgG Titers for *T. Gondii* in the TOXO+ group and PANSS Total and Negative. IgG titers were positively correlated to PANSS Total (**A**) and PANSS negative (**B**). Inserts shows r and p values of the spearman correlation (two-tailed). PANSS Positive and Negative Syndrome Scale.

At baseline, no significant differences between TOXO+ and TOXO− groups were found for positive, negative, general, or total PANSS symptoms, nor for Hamilton Depression and Anxiety scales (Table 1). Then we studied the association between the concentration of antibodies against *T. gondii* and the intensity of schizophrenia symptoms and found significant associations between IgG titers and PANSS Negative (*r* = 0.42, *p* = 0.04) and total (*r* = 0.40, *p* = 0.04) scores (Fig. 2; Supplementary Table 1). Since age and illness duration can impact symptom severity, we tested for associations between those two variables and IgG titers, and found no significant correlations (age: *r* = −0.28, *p* = 0.16; illness duration: *r* = −0.32, *p* = 0.22).

### Response to neuroscience-informed cognitive training in TOXO+ and TOXO− schizophrenia subjects

#### Training adherence

Of the 35 TOXO− individuals with schizophrenia assessed at baseline, 9 dropped out at the first stage of the intervention (25,7%), doing less than 20 h of training; 2 subjects left the intervention after 20 h of training (5,7%), and 24 completed 40 h of training (68,6%) (Supplementary Fig. 1). From the 25 TOXO+ individuals, 1 subject dropped out after 20 h of training (4%), and 24 subjects completed 40 h of training (96%). Chi-squared tests revealed significant differences between adherence to the intervention measured by the intervention stage accomplishment (*X*^*2*^ = 6.92, *p* = 0.03).

Thereafter, to better understand the profile of the dropouts, we compared the subjects who completed (*n* = 42) and did not complete (*n* = 12) the digital cognitive training. We found that the subjects who dropped out of the intervention were significantly younger (*X*^*2*^ = 4.25, *p* = 0.03), but no significant differences were found for sex, education, IQ, years of illness, or chlorpromazine equivalent (Supplementary Table 2). We also found that subjects who completed the intervention had higher z-scores at baseline in the social cognition composite than those who dropped out (*t* = 2.1, *p* = 0.03), even after including age as a covariate (*F* = 1.99, *p* = 0.05). No significant difference was found for any other cognitive domain or clinical measure (Supplementary Table 2).

#### Changes in cognition

To study the association between *T. gondii* seropositivity and changes in cognition after digital cognitive training, we studied only subjects who completed the entire intervention (24 TOXO+ and 24 TOXO− subjects). In this reduced sample, there was no significant difference between groups in age, sex, education, IQ, years of illness, or chlorpromazine equivalent (Supplementary Table 3). Nevertheless, the TOXO+ group showed significantly lower z-scores in attention in comparison to the TOXO− (*t* = 2.10, *p* = 0.04). We also found trends towards significance for lower working memory, verbal memory and learning, and global cognition in the TOXO+ group (Table 2). The changes in cognition after training were similar between TOXO+ and TOXO−groups (Table 2).

**Table 2:** Baseline and endpoint cognitive and clinical data of the TOXO− and TOXO+ patients who completed the digital cognitive training.

	TOXO− (*n* = 24)			TOXO+ (*n* = 24)			Baseline comparison	Changes’ comparison
	baseline	endpoint	change	baseline	endpoint	change		
	Mean (SD)						*t* or *x*^2^ (*p*)^a^	
Cognition (z-scores)								
Speed of processing	−1.2 (2.4)	−1.7 (2.5)	−0.5 (2.3)	−1.7 (2.4)	−1.6 (2.0)	−0.1 (1.9)	0.55 (0.5)	0.14 (0.7)
Attention	−1.0 (1.1)	−0.5 (0.9)	0.4 (0.7)	−1.8 (1.5)	−1.1 (1.3)	0.7 (1.1)	**2.10 (0.04)**	−0.81 (0.4)
Working memory	0.0 (1.1)	0.0 (0.7)	0.0 (0.8)	−0.5 (0.9)	−0.4 (1.0)	0.1 (0.8)	*1.81 (0.08)*	−0.42 (0.7)
Verbal memory and learning	0.1 (1.1)	0.0 (1.1)	−0.1 (0.9)	−0.4 (1.0)	−0.3 (1.0)	0.1 (0.7)	*1.79 (0.08)*	−0.85 (0.4)
Visual memory and learning	−1.5 (1.5)	−1.0 (1.7)	0.5 (1.4)	−1.8 (1.7)	1.4 (1.6)	0.4 (1.4)	0.54 (0.6)	0.22 (0.8)
Reasoning and problem solving	−0.1 (0.6)	0.3 (0.5)	0.4 (0.5)	−0.1 (0.8)	0.3 (0.3)	0.4 (0.7)	0.52 (0.46)	0.28 (0.6)
Social cognition	−0.3 (0.7)	−0.2 (0.7)	0.1 (0.4)	−0.6 (0.5)	−0.2 (0.7)	0.3 (0.6)	1.35 (0.18)	−1.23 (0.2)
Global cognition	−0.6 (0.7)	−0.5 (0.7)	0.1 (0.4)	−1.0 (0.8)	−0.7 (0.7)	0.3 (0.5)	*1.86 (0.07)*	−1.68 (0.1)
Symptoms (raw data)								
HAM-D	6.5 (5.4)	4.9 (3.7)	−1.6 (5.0)	6.2 (5.4)	6.2 (5.0)	0.2 (4.2)	0.17 (0.8)	−1.03 (0.3)
HAM-A	6.6 (5.4)	5.6 (5.1)	−1.0 (6.5)	7.2 (6.9)	6.1 (6.3)	−1.1 (5.5)	−0.29 (0.7)	0.07 (0.9)
PANSS Positive	14.0 (4.9)	11.9 (4.9)	−1.5 (3.2)	13.4 (4.9)	10.3 (3.6)	−2.9 (3.5)	0.45 (0.6)	1.44 (0.1)
PANSS Negative	17.2 (6.6)	16.3 (7.9)	−0.5 (6.3)	14.3 (4.7)	14.2 (5.0)	−0.8 (4.7)	1.64 (0.1)	0.14 (0.9)
PANSS General	29.7 (8.9)	26.2 (5.4)	−3.2 (7.0)	27.1 (5.4)	25.6 (6.4)	−1.5 (5.7)	1.20 (0.2)	−0.90 (0.3)
PANSS Total	61.0 (17.4)	54.4 (12.6)	−5.3 (11.8)	55.0 (12.1)	51.0 (9.8)	−4.4 (8.3)	1.36 (0.1)	−0.30 (0.7)

^a^Kruskal Wallis equality-of-populations rank test was used to compare speed of processing and reasoning and problem-solving z-scores, which did not present normal distribution. All the other variables had normal data distribution and were compared using a *t*-test (two-tailed). *HAM-D* Hamilton Depression Rating Scale, *HAM-A* Hamilton Anxiety Rating Scale, *PANSS* Positive and Negative Syndrome Scale.

Bold values show *p* values < 0.05.

Italic numbers trend towards significance.

#### Changes in schizophrenia symptoms

We compared the changes in schizophrenia symptoms measured by the PANSS scale, and in depressive and anxiety symptoms measured by the Hamilton Scale after digital cognitive training between TOXO+ and TOXO− subjects. We found no significant differences between groups on changes in clinical measures after training (Table 2).

#### Differences in the sensory-modality trained

Finally, we compared the TOXO+ and TOXO− subjects randomized to perform 40 h of either visual or auditory equivalent cognitive exercises, and additionally divided subjects into four groups: TOXO− visual (*n* = 11), TOXO+ visual (*n* = 13), TOXO− auditory (*n* = 13) and TOXO+ auditory (*n* = 11) training. Independent-samples Kruskal-Wallis tests showed that changes in cognition and symptoms after training did not significantly differ between groups (Supplementary Table 4).

## Discussion

In this study, we explored the relationship between seropositivity to *T. gondii* and cognition and symptoms and their changes after digital neuroscience-informed cognitive training in subjects with schizophrenia. The main findings were that TOXO+ subjects presented worse global cognition, with impairments in social cognition and verbal learning. Additionally, we found that TOXO+ subjects showed higher adherence to the digital cognitive training, although changes in cognition and symptoms after training were similar between groups.

We confirmed previous findings of an association between seropositivity to *T. gondii* and lower cognitive performance in schizophrenia subjects. *T. gondii* infection has been repeatedly associated with cognitive decline and other behavioral changes in humans^21,31^, and in animal studies^22^. Regarding its association with schizophrenia, two studies have found lower cognition in chronically infected male subjects with the same serological profile (*T. gondii* IgG+ and IgM-)^24,32^. It is noteworthy that the lower cognition presented by TOXO+ subjects in the present study was maintained after including age as a covariate, an essential step since age increases the probability of having previous contact with the parasite^33^ and is axiomatically associated with cognitive decline.

The TOXO+ group presented higher adherence to treatment (96%) when compared to the TOXO− (68,6%), and subjects who dropped out were younger and had worse social cognition. A recent meta-analysis showed that training social cognition benefits people with schizophrenia on a variety of social-cognitive outcomes^34^. Also, it was found that the addition of social cognition training augmented the response to computer-assisted cognitive remediation for schizophrenia^35^, while the social training alone was associated with gains in social functioning and motivation^36^. Similarly, we observed that baseline social cognition (and age) may impact the adherence to digital cognitive training in schizophrenia.

Despite the lower initial cognition in the TOXO+ group, improvement levels were surprisingly similar between groups after training. These findings contrast with a recent study supporting that worst cognitive performance at baseline predicts larger improvement after digital cognitive training^37^. Here, we showed that TOXO+ subjects were able to highly adhere to the intervention and improve their cognition after 40 h of digital cognitive training.

Unexpectedly, we did not find differences between TOXO+ and TOXO− subjects in schizophrenia symptoms. It has been evidenced that *T. gondii* infection is associated with psychiatric symptoms such as suicide attempts in younger schizophrenia subjects^38^. Similarly, a previous study found that TOXO+ subjects with schizophrenia presented higher PANSS negative scores, including two specific symptoms (delusion and alogia)^27^. Noteworthy, we found a positive association between IgG titers in the TOXO+ group and schizophrenia symptoms (PANSS Negative and Total scores). This result contrasts with a previous study that found an association between lower IgG titers and higher PANSS positive, negative, and disorganized psychopathology scores^32^. However, the higher levels of exposure and the greatest genetic diversity of *T. gondii* strains in Brazil, in comparison with Europe and United States^32^, must be considered when comparing *T. gondii* antibody titers from Brazilian cohorts to others. In fact, high rates of exposure and reinfection in Brazil can cause the elevation and maintenance of high anti-T. *gondii* IgG titers^39–42^.

Furthermore, we investigated the impact of the sensory modality trained on the response to treatment, comparing the changes in cognition and symptoms between TOXO+ and TOXO− subjects randomized to either visual or auditory equivalent cognitive exercises. The crucially decreased sample size in this analysis probably prevented finding any potentially significant differences between groups. In a prior report, our research group specifically explored the differences between visual and auditory exercises in a larger sample of schizophrenia subjects and found that both sensory modalities were effective, but in different ways: the visual group had broader cognitive gains and symptoms improvement than the auditory one^7^, and the auditory group appeared to be more efficacious in the enhancement of emotion processing and social cognition^43^.

The underlying mechanisms of the association between *T. gondi*i seropositivity and cognitive and behavioral alterations remain relatively unknown^44^. A possible biological explanation is an immune-mediation response by proinflammatory biomarkers^45,46^. *T. gondii* is the infectious agent most associated with cognitive impairment in subjects with schizophrenia^13^. Its mechanisms involve dysregulation in dopamine metabolism^47,48^, glutamate synaptic neurotransmission^49^, such as dysfunction in NMDA receptor (NMDAR) with a production of NMDAR antagonist and immune cross-reactions with the receptor^50–54^. These brain alterations seem to be related to cognitive impairment, as demonstrated by a study showing that individuals seropositive for both *T. gondii* and NMDAR antibodies had a decreased performance on the delayed memory module of Repeatable Battery for the Assessment of Neuropsychological Status (RBANS)^55^.

Considering that *T. gondii* infection has been repeatedly associated with schizophrenia^56^, animals infected by *T. gondii* have been used as models to further understand the pathophysiology of schizophrenia^49^. Our research group recently observed severe neural damage induced by chronic infection of C57Bl/6 mice with the ME-49 *T. gondii* strain, with reduced glutamate and D-serine levels in prefrontal cortical and hippocampal tissue homogenates^57^. Other murine studies have shown that the parasite induces impairments in learning and memory, and more particularly in short-term social recognition memory^58^. It has also been demonstrated that *T. gondii* may lead to changes in behavior, leading animals to feel attracted, rather than repulsed by their predators^59^.

*T. gondii* transmission can occur in many ways (i.e., consuming water contaminated with oocysts), and its prevention consists mostly of hygienic-sanitary measures^60^. Since better socioeconomic status is associated with better cognition^30^, it is important to analyze determinants of socioeconomic vulnerability that are associated with exposure to the parasite^29^. Here, we analyzed the Social Development Index, which includes indicators such as sanitation and housing quality, and observed no significant difference between the TOXO+ and TOXO− groups. Thus, we confirmed that the differences in cognition between TOXO+ and TOXO− subjects are likely not to be explained by differences in socioeconomic status.

Limitations of this study include a relatively small sample size (*n* = 60), especially after the intervention (*n* = 48). We had a 20% dropout rate after 40 h of digital cognitive training, which occurred almost exclusively in the TOXO− group (18%), limiting our conclusions on the effects of the intervention. The dropouts were younger TOXO− subjects with lower social cognition. This issue could be addressed in future studies by including targeted social-cognitive training and selecting a dosage of training that would be more engaging to younger individuals with schizophrenia. Moreover, we did not control for the parasite strains. Given that the *T. gondii* population in Brazil is highly diverse, and that its prevalence is different depending on the geographic location^61^, a national multi-sample study would be necessary to compare cognitive impairment in subjects with schizophrenia caused by distinct strains of the parasite in different regions of Brazil.

In conclusion, the present findings highlight the association between *T. gondii* infection and worse cognition in subjects with schizophrenia. Despite the lower cognitive performance at baseline, seropositivity was associated with higher adherence to the digital cognitive training and similar changes in cognition and symptoms after training. These findings highlight the potential of digital cognitive training to remediate cognition in schizophrenia individuals seropositive to *T. gondii*.

## Methods

### Participants

Individuals with chronic schizophrenia or schizoaffective disorder were recruited in the context of a randomized, double-blind clinical trial on neuroscience-informed digital cognitive training (details of the study are fully described in our previous publication^7^). Subjects were recruited from the day-hospital and outpatient clinic of the Institute of Psychiatry (IPUB) at the Federal University of Rio de Janeiro (UFRJ) from September 2013 to December 2016.

Participants were included if they were between 18 and 60 years of age, had a diagnosis of schizophrenia or schizoaffective disorder, had an IQ above 80^62^, were clinically stable, and had an outpatient status for at least one month before starting the intervention. Participants were excluded if they were illiterate, or had any history of another psychiatric diagnosis, intellectual disability, or brain damage. All participants signed a written consent form after being informed about the study procedures. The study was approved by the Brazilian National Committee of Ethics in Research (12990013.0.0000,5263) and pre-registered at ClinicalTrials.gov (1R03TW009002-01).

### Study design

This was a randomized, double-blind, parallel-design study in which participants performed 40 h of auditory versus visual neuroscience-informed digital cognitive training. Blood samples were collected at baseline and cognitive functions and clinical symptoms were assessed at baseline and after 40 h of training. After the intervention, we divided participants into TOXO+ (IgG + ) and TOXO− (IgG-) groups.

### Assessments

#### Cognition

Seven cognitive domains have been defined as impaired in schizophrenia by the Measurement and Treatment Research to Improve Cognition in Schizophrenia (MATRICS). We used the MATRICS Consensus Cognitive Battery recommended tests (MCCB) and the Cambridge Neuropsychological Test Automated Battery (CANTAB) to assess speed of processing (Category Fluency (CF) and Reaction time (RTI) tests), attention (Rapid Visual Processing (RVP) test), working memory (digit backward (DB) and Spatial Working Memory (SWM) tests), verbal learning (Hopkins Verbal Learning Task (HVLT)), visual learning (Brief Visuospatial Memory Test (BVMT)), reasoning and problem solving (Stocking Of Cambridge (SOC) test), and social cognition (Mayer-Salovey-Caruso Emotional Intelligence Test – Managing emotions (MSCEIT). A fully detailed explanation of these tests can be found in previous studies^63,64^. Global cognition was a composite score calculated from the seven cognitive domain scores mentioned above.

#### Symptoms

The Positive and Negative Syndrome Scale (PANSS)^37^, and the Hamilton Depression^65^ and Anxiety^66^ rating scales were administered at baseline and after the intervention to assess participants’ clinical status.

#### Measurement of Socioeconomic Status (SES)

To measure socioeconomic status, we used the Social Development Index^67^, which analyzes the urban situation and the socio-economic status of the neighborhoods in Rio de Janeiro, Brazil. The index is calculated considering 4 indicators: access to basic sanitation (including adequate water supply, garbage collection, and sewage services), housing quality, degree of education, and the average income of each house. The result is the average of normalized values from these indicators, ranging from 0 to 1 (higher values indicate greater social development). Data was provided by Rio de Janeiro’s city hall.

### Neuroscience-informed cognitive training

Participants were required to practice the neuroscience-informed cognitive training exercises for 1 h daily, 3 to 5 times a week until they completed 40 h of training. The digital cognitive training consisted of two groups (auditory and visual) of six exercises that trained speed processing, attention, memory, working memory, executive function, and social cognition in the selected perceptual modality. The two groups’ exercises had equivalent task dynamics and were conceived to adapt their difficulty levels according to participants’ performance^8^. An algorithm was used to maintain performance at rates of 80% of success, in order to keep participants engaged in the task and correct responses were rewarded through sounds, fireworks, and the accumulation of stars. Further details about the cognitive training exercises are described in Scoriels et al. 2020. The exercises were provided by *Posit Science, Inc*. (www.brainhq.com).

### *T. gondii* antibody levels measurement

Venous blood samples were collected from subjects selected at baseline and stored at −80 °C until analysis. *T. gondii* tachyzoite (RH strain) suspension was prepared following instructions described in^68^. To assess immunoglobulin production, fixed *T. gondii* tachyzoites were used in a flow cytometry-based assay. IgM and IgG titers were measured in the 1:16000 dilution with a cutoff of 80% for IgM and 20% for IgG, and experiments were performed in 96-well plates with a slightly modified protocol compared with the one described in^68^. More detailed information regarding blood collection and processing, tachyzoite preparation, and immunofluorescence by flow cytometry can be found in the Supplementary Methods.

### Data analysis

The distributions of demographic, cognitive, and clinical data were tested for normality using the Shapiro-Wilk Test. First, we compared the TOXO+ and TOXO− groups at baseline using Independent Samples t-tests (two-tailed) or Kruskal-Wallis equality-of-populations rank test, depending on the normality and homogeneity of distributions. Since age was significantly different between groups at baseline, we included this variable as a covariate using General Linear Models when the assumptions were met (i.e., homoscedasticity and normality of the residuals), and Quantile Regression Models^69^ when those assumptions were not met. Next, we conducted Spearman correlations in the TOXO+ group at baseline to test the association between the IgG Titers to *T. gondii* and cognition and schizophrenia symptoms.

We further performed a Chi-square test to compare the adherence to the intervention between TOXO+ and TOXO− subjects. In the post-intervention analysis, we only included subjects who completed the cognitive training. We retested the normality of data distributions with the Shapiro-Wilk test, and differences in baseline characteristics, as well as changes in cognition and symptoms (endpoint minus baseline scores) using Independent Samples t-tests (two-tailed) or Kruskal-Wallis equality-of-populations rank test. To answer whether the sensory-modality training had an impact on patients’ responses, we performed Kruskal Wallis comparisons between the four groups (TOXO+ visual, TOXO− visual, TOXO+ auditory, and TOXO− auditory). We also compared participants who completed and did not complete the intervention testing for differences in demographic, cognitive, and clinical data using Independent Samples t-tests (two-tailed) or Kruskal-Wallis equality-of-populations rank test.

We used participants’ neuropsychological tests z-scores or z-score change (further details are described in our previous publication^7^, and the raw data of the clinical scales. Data were analyzed using IBM SPSS (28.0 version) and STATA (Version 8.0) Software, with a statistical significance level set at *p* < 0.05.

## Supplementary information

Supplementary Figure 1

Supplementary Table 1

Supplementary Table 2

Supplementary Table 3

Supplementary Table 4

## Data Availability

The data that support the findings of this study are available from the corresponding author upon reasonable request.
