# Author Correction: Effects of *Toxoplasma gondii* infection on cognition, symptoms, and response to digital cognitive training in schizophrenia

**DOI:** 10.1038/s41537-022-00327-8

**Published:** 2022-12-30

**Authors:** Anna Luiza Guimarães, David Richer Araujo Coelho, Linda Scoriels, Juliana Mambrini, Lis Ribeiro do Valle Antonelli, Priscilla Henriques, Andréa Teixeira-Carvalho, Olindo Assis Martins Filho, José Mineo, Lilian Bahia-Oliveira, Rogério Panizzutti

**Affiliations:** 1 Instituto de Psiquiatria, Universidade Federal do Rio de Janeiro, Rio de Janeiro, Brazil; 2 Instituto de Ciências Biomédicas, Universidade Federal do Rio de Janeiro, Rio de Janeiro, Brazil; 3 Departamento de Imunoparasitologia, Universidade Federal do Rio de Janeiro, Rio de Janeiro, Brazil; 4 Instituto René Rachou, Fundação Oswaldo Cruz, Rio de Janeiro, Brazil; 5 Universidade Federal de Uberlândia, Uberlândia, Brazil

**Keywords:** Schizophrenia, Learning and memory

Correction to: *Schizophrenia* 10.1038/s41537-022-00292-2, published online 25 November 2022

In this article the authors’ names David Richer Araujo Coelho, Lis Ribeiro do Valle Antonelli, and Olindo Assis Martins Filho were incorrectly listed as David Coelho, Lis Antonelli, and Olindo Martins-Filho. The original article has been corrected.

