# Supplementary material for: Effects of Toxoplasma gondii infection on cognition, symptoms, and response to digital cognitive training in schizophrenia
Source: Schizophrenia (Heidelb). 2022 Nov 25;8(1):104. doi: 10.1038/s41537-022-00292-2 (PMC9700796; doi:10.1038/s41537-022-00292-2)
Supplement: Supplementary file 1 — Supplementary Figure 1 [file 41537_2022_292_MOESM1_ESM.pdf]

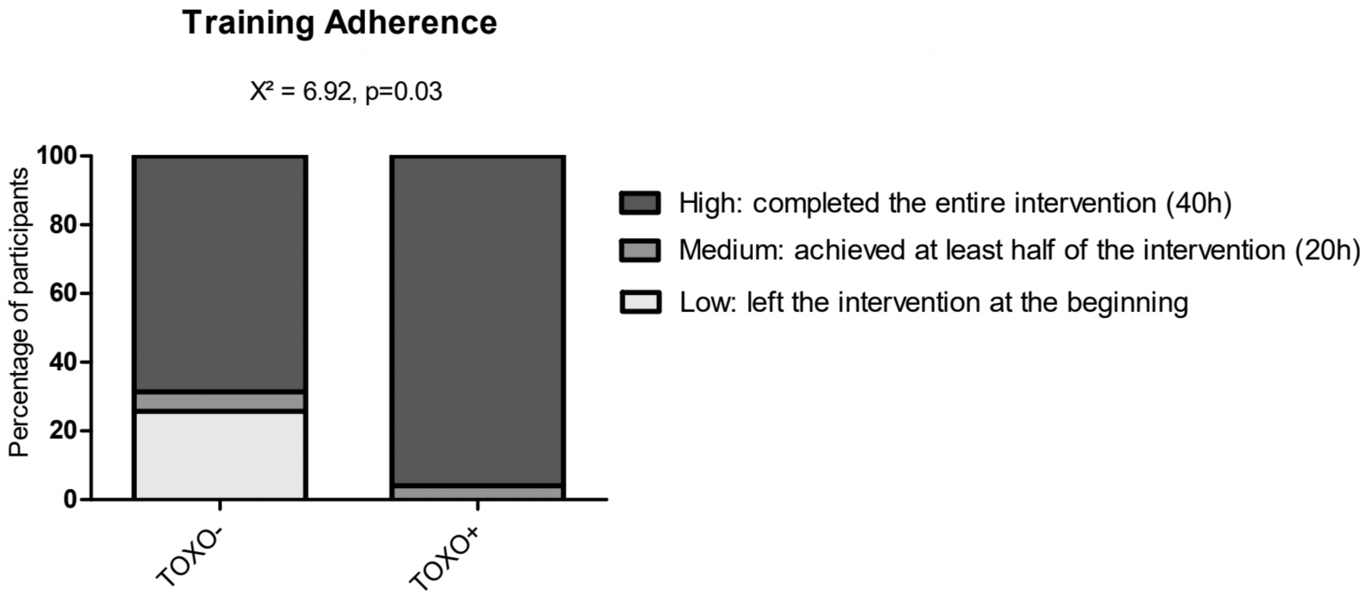

Supplementary Figure 1. The adherence to digital cognitive training was defined by the completed training stages.
Insert shows Chi-squared test results, revealing a significant difference between groups.
