# Supplementary material for: Effects of Toxoplasma gondii infection on cognition, symptoms, and response to digital cognitive training in schizophrenia
Source: Schizophrenia (Heidelb). 2022 Nov 25;8(1):104. doi: 10.1038/s41537-022-00292-2 (PMC9700796; doi:10.1038/s41537-022-00292-2)
Supplement: Supplementary file 2 — Supplementary Table 1 [file 41537_2022_292_MOESM2_ESM.pdf]

Supplementary Table 1. Spearman correlations between IgG antibodies and cognitive and clinical measures in the TOXO+ group.

	IgG Titers	
	ρ	p-value
Cognition		
Speed of processing	-0.09	0.66
Attention	-0.30	0.14
Working memory	-0.23	0.26
Verbal learning	-0.30	0.14
Visual learning	-0.07	0.71
Reasoning and problem solving	0.08	0.69
Social cognition	-0.39	0.07
Global cognition	-0.26	0.20
Clinical Symptoms		
HAM-D	0.14	0.50
HAM-A	-0.09	0.65
PANSS Positive	0.20	0.34
PANSS Negative	**0.42**	**0.04**
PANSS General	0.30	0.15
PANSS Total	**0.40**	**0.04**

HAM-D: Hamilton Depression Rating Scale;
HAM-A: Hamilton Anxiety Rating Scale;
PANSS: Positive and Negative Syndrome Scale.
