# Supplementary material for: Effects of Toxoplasma gondii infection on cognition, symptoms, and response to digital cognitive training in schizophrenia
Source: Schizophrenia (Heidelb). 2022 Nov 25;8(1):104. doi: 10.1038/s41537-022-00292-2 (PMC9700796; doi:10.1038/s41537-022-00292-2)
Supplement: Supplementary file 3 — Supplementary Table 2 [file 41537_2022_292_MOESM3_ESM.pdf]

Supplementary Table 2. Comparison of baseline characteristics of subjects who completed versus did not complete the digital cognitive training.

	Completed (n=48)	Did not complete (n=12)	t or x² (p)[a]
	Mean (SD)	Mean (SD)	
**Age (years)**	41 (11)	34 (11)	**4.25 (0.03)**
Female/male	33/15	8/4	0.19 (0.57)
Education (years)	11 (3)	12 (0.8)	-1.08 (0.28)
IQ	101 (13)	106 (16)	-1.37 (0.17)
Years of Illness	16 (11)	18 (11)	0.51 (0.47)
CPZ equivalent (mg)	481 (425)	517 (317)	0.50 (0.47)
Training type (visual/auditory)	23/25	7/5	0.41 (0.37)
Baseline cognition (z-scores)			
Speed of processing	-1.49 (2.41)	-0.80 (1.60)	0.54 (0.4)
Attention	-1.43 (1.45)	-0.85 (1.20)	-1.27 (0.2)
Working memory	-0.23 (1.02)	-0.36 (1.57)	0.33 (0.7)
Verbal learning	-0.17 (1.09)	0.17 (1.39)	-0.9 (0.3)
Visual learning	-1.66 (1.57)	1.00 (1.03)	-1.3 (0.1)
Reasoning and problem solving	0.11 (0.72)	0.05 (0.53)	0.34 (0.5)
Social cognition	0.48 (0.70)	0.00 (0.67)	**2.1 (0.03)**
Global cognition	-0.81 (0.82)	-0.39 (0.83)	-1.5 (0.1)
Baseline clinical measures (range)			
HAM-D (0-54)	6 (5)	7 (7)	0.0 (0.9)
HAM-A (0-56)	7 (6)	7 (7)	0.1 (0.7)
PANSS Positive score (7-49)	13 (4)	13 (5)	0.1 (0.9)
PANSS Negative score (7-49)	15 (5)	15 (7)	0.0 (0.9)
PANSS General score (16-112)	28 (7)	28 (5)	0.0 (0.9)
PANSS Total Score (30-210)	58 (15)	58 (14)	0.1 (1.0)

HAM-D: Hamilton Depression Rating Scale; HAM-A: Hamilton Anxiety Rating Scale PANSS: Positive and Negative Syndrome Scale.

[a]: kruskal wallis equality-of-populations rank test was used to compare speed of processing, reasoning and problem solving z-scores, and for HAM-D and AM-A, which did not present normal distribution. All the other variables had normal data distribution and were compared using a t-test (two-tailed).

[b]: Since that was an age difference between groups, we tested this difference with a general linear model including age as a covariate, and the difference was mantained (F=1.99, p=0.05).
