# Supplementary material for: Effects of Toxoplasma gondii infection on cognition, symptoms, and response to digital cognitive training in schizophrenia
Source: Schizophrenia (Heidelb). 2022 Nov 25;8(1):104. doi: 10.1038/s41537-022-00292-2 (PMC9700796; doi:10.1038/s41537-022-00292-2)
Supplement: Supplementary file 4 — Supplementary Table 3 [file 41537_2022_292_MOESM4_ESM.pdf]

Supplementary Table 3. Participants baseline charateristics including only the subjects who finished the digital cognitive training.

	TOXO- (n=24)	TOXO+ (n=24)	Statistics
	Mean (SD)		t or X^2 (p)[a]
Age (years)	38 (12)	44 (8)	2.36 (0.12)
Female/male	7/17	8/16	0.09 (1.00)
Education (years)	12 (3)	12 (3)	1.58 (0.20)
IQ	104 (11)	94 (13)	2.03 (0.15)
Years of Illness	12 (10)	18 (11)	2.12 (0.14)
CPZ equivalent (mg)	324 (367)	320 (488)	0.01 (0.91)
