# Supplementary material for: Effects of Toxoplasma gondii infection on cognition, symptoms, and response to digital cognitive training in schizophrenia
Source: Schizophrenia (Heidelb). 2022 Nov 25;8(1):104. doi: 10.1038/s41537-022-00292-2 (PMC9700796; doi:10.1038/s41537-022-00292-2)
Supplement: Supplementary file 5 — Supplementary Table 4 [file 41537_2022_292_MOESM5_ESM.pdf]

Supplementary Table 4. Changes after digital cognitive training among TOXO+ and TOXO- groups, divided by sensory modality trained

	Visual training		Auditory training		
	TOXO- (n=11)	TOXO+ (n=13)	TOXO- (n=13)	TOXO+ (n=11)	Statistics
	Mean (SD)	Mean (SD)	Mean (SD)	Mean (SD)	x^2 (p)
Changes in cognition (z-scores)					
Speed of processing	-0.44 (0.91)	-0.24 (0.33)	-0.7 (0.30)	-0.06 (0.95)	0.43 (0.93)
Attention	0.29 (0.23)	1.28 (0.46)	0.49 (0.21)	0.34 (0.45)	3.62 (0.30)
Working memory	0.37 (0.24)	0.54 (0.24)	-0.30 (0.27)	-0.16 (0.19)	3.88 (0.27)
Verbal learning	-0.08 (0.39)	0.32 (0.21)	-0.13 (0.25)	0.12 (0.33)	0.99 (0.80)
Visual learning	0.33 (0.34)	1.10 (0.38)	0.41 (0.49)	-0.20 (0.37)	5.79 (0.12)
Reasoning and problem solving	0.26 (0.22)	-0.04 (0.13)	0.48 (0.12)	0.40 (0.15)	3.19 (0.36)
Social cognition	0.00 (0.14)	0.15 (0.22)	0.04 (0.96)	0.51 (0.15)	3.34 (0.34)
Global cognition	0.04 (0.19)	0.4 (0.16)	0.13 (0.07)	0.13 (0.17)	5.20 (0.15)
Changens in symptoms (raw data)					
PANSS Positive	-1.5 (1.35)	-3.57 (1.5)	-1.00 (0.76)	-2.33 (1.39)	1.74 (0.62)
PANSS Negative	-1.77 (2.05)	-4.42 (1.71)	0.83 (1.30)	-0.22 (1.13)	2.09 (0.55)
PANSS General	-2.33 (0.92)	-2.85 (1.85)	-2.08 (2.35)	-1.55 (2.45)	0.82 (0.84)
PANSS Total	-5.66 (2.97)	-10.85 (2.89)	-2.25 (3.22)	-1.88 (2.88)	1.31 (0.72)
